# The impact of loco-regional anaesthesia on postoperative opioid use in elderly hip fracture patients: an observational study

**DOI:** 10.1007/s00068-021-01674-4

**Published:** 2021-05-07

**Authors:** Gioia Häusler, Puck C. R. van der Vet, Frank J. P. Beeres, Thomas Kaufman, Jip Q. Kusen, Beate Poblete

**Affiliations:** 1 Department of Anesthesiology, Luzerner Kantonsspital, Luzern, Switzerland; 2 Department of Orthopaedic and Trauma Surgery, Luzerner Kantonsspital, Luzern, Switzerland; 3 Minusio, Switzerland

**Keywords:** Loco-regional anaesthesia, Elderly hip fracture patients, Geriatric trauma care

## Abstract

**Purpose:**

Hip fractures are a common health problem among the elderly with an increasing incidence. They are associated with high mortality and morbidity. Optimal pain management remains challenging and inadequate pain control is known for negatively affecting outcomes. Loco-regional anaesthetics (LRA) have been proven to benefit pain management and to lower the risks of opioid use and -related side effects. We aimed to evaluate the use and efficacy of different LRA in elderly hip fracture patients.

**Methods:**

Single-center cohort study of elderly hip fracture patients, who were treated in central Switzerland. We compared patients who received LRA in the form of a femoral nerve block (FNB) or a continuous femoral nerve catheter (CFNC) with patients who did not receive LRA. Primary outcomes were pain—as measured in perioperative morphine use—hospital length of stay (HLOS), postoperative complications, postoperative falls and mortality.

**Results:**

407 patients were included for analysis. Mean age was 85.2 (SD6.3). There was a significant difference in intraoperative morphine use between the groups (*p* = 0.007). Postoperative morphine use differed significantly and was lowest in patients with FNB and highest in patients without LRA (*p* < 0.001). The use of LRA was a significant predictor for postoperative morphine use for postoperative morphine use at the recovery room and for postoperative morphine use 48 h after surgery. No significant differences were found in postoperative complications, a significant difference was found in 1-year mortality.

**Conclusions:**

This article shows that LRA in the form of FNB and CFNC causes a significant decrease in postoperative opioid consumption. Differences between single-shot FNB or CFNC were minimal. There were no significant differences in clinical outcomes such as HLOS, delirium, 30-day and 90-day mortality and postoperative falls. We suggest that use of LRA should be incorporated in the perioperative treatment of elderly patients with a hip fracture. For future research, we recommend evaluating the number of postoperative complications and mortality.

**Supplementary Information:**

The online version contains supplementary material available at 10.1007/s00068-021-01674-4.

## Introduction

Hip fractures in elderly patients are the most common type of fracture and a well-known public health problem [1]. They are associated with high morbidity, mortality and costs [2, 3]. Moreover, the incidence of hip fractures increases with age. Therefore, due to the current aging population, the number of patients with a hip fracture is expected to increase even further [4, 5]. Patients with a hip fracture often report severe pain and previous literature shows that inadequate pain control can negatively affect outcomes after a hip fracture. [3, 6, 7] For this reason, optimal pain management is of upmost importance in elderly patients with a hip fracture.

Nonetheless, determining optimal pain management in this patient population, in which comorbidities and polypharmacy are common, remains challenging [8]. Recent literature describes the potential benefits of loco-regional anaesthesia (LRA) in the form of a single-shot femoral nerve block (FNB) and a continuous femoral nerve catheter (CFNC) [7, 9–11]. Before the introduction of these loco-regional anaesthetic interventions, patients received only parenteral or oral opioids. These opioids are known for their side effects, the most common of which are nausea/vomiting, addiction, delirium and respiratory depression. [3, 7] LRA like CFNC and FNB have been proven to be effective in limiting opioid use and, therefore, opioid-related side effects, however, despite these benefits, previous literature shows that LRA are still not commonly used in hip fracture patients [12]. Most studies focus on either CFNC or FNB and there is a dearth of studies reviewing both manners of LRA concurrently [9, 13–16].

At a level-1 trauma center in Central Switzerland, a geriatric fracture centre (GFC) was recently implemented to optimize surgical treatment in elderly (hip) fracture patients. An important aspect of the GFC was to optimize anaesthesia. This study was based on a quality assessment and aimed to assess the quality and use of (loco-regional) anaesthesia in hip fracture patients treated in the Luzerner Kantonsspital (LUKS). Therefore, we evaluated perioperative pain control—as measured by the opioid use—in elderly hip fracture patients with LRA (in the form of FNB or CFNC) versus elderly hip fracture patients without LRA. The secondary aim was to evaluate the number of postoperative complications (e.g., falls and delirium diagnoses), postoperative 30-day and 90-day and 1-year mortality and to observe potential differences between the forms of anaesthesia (FNB, CFNC or no LRA). We hypothesized that use of LRA would lead to fewer opioid use.

## Methods

This article is written in accordance with the STROBE statement [17].

A single-center observational cohort study based on quality assessment on the anaesthetic protocol of elderly hip fracture patients was conducted at the largest trauma center of Central Switzerland. This study was based on data that were collected for quality improvement purposes. Ethical approval for the quality improvement project was given by the responsible ethical commission/ Institutional Review Bord (IRB) (Ethikkommission Nordwest- und Zentralschweiz, Hebelstrasse 53, Basel, Switzerland, President Prof. A.P. Perruchoud, EKNZ 2014–343). Since this was considered a quality assessment, the IRB waived the requirement for written informed consents.

All patients, who were 70 years of age or older, presenting to the hospital with a proximal femur fracture, between 01.01.2015 and 07.30.2017 who received operative treatment were included for analysis. Fractures were classified using the Arbeitsgemeinschaft für Osteosynthesefragen (AO) fracture classification system. [18] Patients who postoperatively received Patient Controlled Analgesia (PCA) were excluded from analysis because the exact dose of administered pain medication could not be monitored adequately. Other exclusion criteria were patients who did not have available data or patients who did not live in Switzerland (wherefore, no follow-up data could be collected). All patient data at the hospital are uniformly recorded and documented in the hospital database, including patient demographic information, diagnoses at admission and laboratory results. Daily progress summaries and discharge letters (including information such as hospital length of stay (HLOS), delirium diagnosis (as measured by the Confusion Assessment Method (CAM)) documented by the responsible medical doctor are also available on this database. Original anaesthetic protocols and admission of anaesthetics of all patients were collected and reviewed. American Society of Anaesthesiologists (ASA) scores were calculated by the attending anaesthetist and confirmed by the anaesthetist managing the quality improvement project [19]. All data were collected retrospectively, data collection started in 2018.

The primary outcome measure was the amount of pain—as measured by opioid use—of all patients. Secondary outcomes included 30-day-, 90-day-, and 1-year mortality and complications, such as HLOS, delirium (as measured by the CAM test), and the number of falls within 48 h after the placement of a block or the catheter. All patients were analysed within three moments in time, namely preoperatively, intraoperatively and postoperatively.

Preoperative data consisted of:

Age, gender and ASA score. Type of intervention was categorised into the three following groups: (i) patients who had not received any LRA (ii) patients who had received FNB and (iii) patients who had received CFNC.

Opioid use at the emergency ward was noted and, in an attempt to make data on opioid use as uniform as possible, we used morphine equivalents, calculated using an equianalgesic calculator. [20]

Intraoperative data were categorised in two ways. First, we observed the whole study population in terms of surgery time, time to surgery, type of surgery received, and type of anaesthetic used (e.g., spinal anaesthetics or general anaesthetics). Hereafter, we divided the patients into the three previously named groups based on intervention. We then evaluated: type of LRA used (e.g., ropivacaine or prilocaine), amount of LRA anaesthetic used (in ml), the number of patients that received a second gift of LRA (e.g., bupivacaine, levobupivacaine hydrochloride, hydromorphone, ropivacaine or prilocaine). The time between admission of the intervention and the start of surgery, the time spent at the recovery room and opioid use (noted in morphine equivalents).

Postoperative data were:

Opioid use during time spent in the recovery room, opioid use 48 h postoperatively, total HLOS, complications (patients who were diagnosed with delirium, myocardial infarction (MI), cerebrovascular accident (CVA), wound infection, urinary tract infection, decubital ulcer and anaemia [all according to the World Health Organization [WHO] guidelines]) [21]. In addition, we collected data on the number of patients who experienced a postoperative fall. Data on 30-day-, 90-day-, and 1-year mortality was collected using national registries.

As for the choice of intervention: preferably, in all patients administered to the emergency ward who required extra analgesia CFNC was the primary choice of LRA. However, if coagulation disorders were noted, FNB was given due to the fact that a single-shot FNB-induced less trauma (and, therefore, a lower risk of bleeding) then a CFNC. If patients suffered from severe coagulation disorders, no LRA was giving due to high risk of bleeding. This was assessed by the anaesthesiologist on duty. If patients had a history of femoral-popliteal bypass or if they had an infection (e.g., cellulitis), no LRA was given as well.

Patients received CFNC or FNB from a fully educated anaesthesiologist and anaesthesiologists in training under supervision. The fully educated anaesthesiologist also made the choice of type of LRA. According to protocol, ultrasounds were used for block placement. Further specifications on placement on block and catheter are found in the hospitals’ protocol. [22]

### Statistical analysis

Descriptive statistics were used for analysing baseline-, peri-, and postoperative data. Means (M) and standard deviations (SD) were calculated for numeric, normally distributed data. Medians (Mdn) and interquartile ranges (Q1:Q3) were calculated for quantitative, skewed data. The Shapiro–Wilk test was used to test for normality. Missing data were excluded from analysis. For calculating the use of opioids, we used the aforementioned opioid equianalgesic calculator to covert opioid consumption to intravenous milligram morphine equivalent [20]. Subgroup analysis were made based on different types of intervention. Multiple linear regression was used to test for predicters in opioid use between the different intervention groups. The effects of the variables age, gender, ASA score, time to surgery and type of surgery were accounted for in the regression model.

The ANOVA test was used to test for mean differences in the use of anaesthetics preoperatively, intraoperatively and postoperatively up to 48 h. The alpha-level for statistical significance was set at 0.05. Analyses were carried out using SPSS Statistics version 25 (IBM Corporation Armonk, NY).

## Results

### Study population

**Fig. 1:**
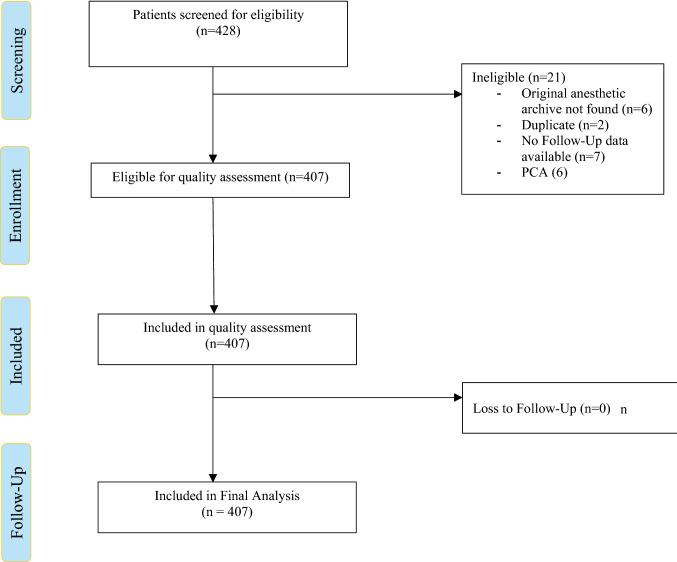
Patient flow chart

In total, 428 patients were admitted with a proximal femur fracture. Of those, 21 were excluded because they did not meet eligibility criteria. Figure 1 shows the patient flowchart and reasons for exclusion. Characteristics of the study population can be seen in Table 1.

**Table 1:** Baseline characteristics

	FNB (*N* = 118)	CFNC (*N *= 176)	No LRA (*N* = 112)	Total	*p*-value
Age (years) mean ± SD	85.8 ± 6.3	85.0 ± 6.2	84.9(6.4)	85.2 ± 6.3	0.464
Gender Male *n* (%) Female * n* (%)	32(27.1) 86(72.9)	43(24.4) 133(75.6)	30(26.8) 82(73.2)	105(25.9) 301(74.1)	0.846
ASA classification					**0.011**
ASA classification 1 * n* (%)	0(0)	0(0)	1(0.9)	1(0.2)	
ASA classification 2 * n* (%)	32(27.1)	32(18.3)	25(22.3)	89(22)	
ASA classification 3 * n* (%)	84(71.2)	119(68.0)	73(65.2)	276(68.1)	
ASA classification 4 * n* (%)	2(1.7)	24(13.7)	13(11.6)	39(9.6)	

*n*: number of patients, *SD*: Standard Deviation, *ASA* *classification* American Society of Anaesthesiologists Physical Status Classification System, *FNB* femoral nerve block, *CNFC* continuous femoral nerve catheter

At presentation, 1 patient was scored ASA 1 (0.2%) and 276 (68.1%) patients received an ASA 3 score. A total of 176 (43.3%) of the 407 included patients received CFNC. 118 patients (29.1%) received FNB and 112 patients (27.6%) received no LRA. A significant difference in ASA score at baseline was found (*p* = 0.011). When comparing only LRA to no LRA, no significant difference in ASA score was found (*p* = 0.327).

### Pre-, intra-, and postoperative outcomes

Mean time to surgery was 23.3 h (SD 4.8) and mean surgery time was 118 min (SD 51.9). Type of surgery performed was hemiarthroplasty in 155 patients (38.3%) and in 164 patients cannulated screws were used (40.4%). Most patients (88.7%, *n* = 360) received general anaesthesia (Table 2). Supplements 2, 3 and 4 show the boxplots of the pre-, intra-, and postoperative morphine use. Perioperative outcomes are shown in Table 3.

**Table 2:** Perioperative outcomes

	FNB (*n* = 116)	CFNC (*n* = 176)	No LRA (*n* = 112)	Total	*p*-value
Surgery duration (minutes) *M (SD)*	110.36(42.8)	122.63(58.5)	115.70(47.5)	117.19(51.6)	0.130
Time to surgery (hours) *M (SD)*	10.21(6.5)	13.53(6.9)	12.93(7.2)	12.40(7)	** < 0.001**
Type of surgery performed					0.225
Hemiarthroplasty *n* (%)	54(45.8)	54(30.7)	47(42.0)	155(38.2)	
DHS *n* (%)	6(5.1)	12(6.8)	6(5.4)	24(5.9)	
Gammanail-TFN *n* (%)	4(3.4)	3(1.7)	5(4.5)	12(3.0)	
Cannulated screws *n* (%)	45(38.1)	78(44.3)	41(36.6)	164(40.4)	
Proximal Femoral Plate *n* (%)	4(3.4)	10(5.7)	3(2.7)	17(4.2)	
THP *n* (%)	2(1.7)	11(6.3)	8(7.1)	21 (5.2)	
Cerclage *n* (%)	0(0.0)	4(2.3)	0(0.0)	4 (1.0)	
Missing *n* (%)	3(2.5)	4(2.3)	2(1.8)	9 (2.2)	
Type of anesthetics used					**0.017**
Spinal *n* (%)	9(7.6)	29(16.5)	8(7.1)	46(11.3)	
General *n* (%)	107(92.4)	147(83.5)	104(92.9)	360(88.7)	

*N* number of patients, *M* Mean, *SD* Standard Deviation, *DHS* Dynamic Hip Screw, *TFN *Trochanteric Fixation Nail,* THP* Total Hip Replacement, *FNB* femoral nerve block, *CNFC* continuous femoral nerve catheter

**Table 3:** Perioperative outcomes and types of anaesthetics used

	FNB (*N* = 118)	CFNC (*N* = 178)	No loco-regional anaesthesia (*N* = 112)	*p*-value
Type of loco-regional anaesthetic used				
Ropivacaine 0.75%/Pirilo1% (1:1) in ml *M (SD)*	21.4 (9.6)	21.2 (7.8)		
Prilocaine 1% in ml *M (SD)*	6.2 (3.8)	7.6(4.2)		
No. of patients receiving second gift of local anaesthetic *N (%)*	14 (11.9)	110 (62.5)		
Second gift of local anaesthetic in ml *M (SD)*	3.2 (9.2)	0.7 (4.6)		
Total volume of local anaesthetic	30.8 (8.3)	29.6 (9.3)		
Time at recovery (hours) *M (SD)*	4:04 (2:44)	5:36 (4:33)	4:43 (4:11)	**0.006**
Time between admission of intervention and surgery (hours) *M (SD)*	1:52 (4:39)	10:50 (8:11)		< 0.001

*n* number of patients, *M* mean, *SD* Standard Deviation

### Opioid use

#### FNB group

Mean opioid use (as measured by the morphine equivalent) preoperatively in the FNB group was 3.5 mg (SD 4.8; 95% CI: 2.7–5.1) (Table 4). Intraoperative use of opioids was 31.1 mg (SD 13.6; 95% CI 28.6–33.6) and postoperatively at the recovery room 1.2 mg (SD 3.4; 95% CI 0.6–1.8) was used and 2.1 mg (SD 5.1; 95% CI 1.2–3.1) 2 days after surgery. Postoperative (both at the recovery room as 48 h after surgery) opioid use in the FNB group was significantly lower compared to the other groups (recovery room: *p* < 0.001; after 48 h: *p* = 0.015). When comparing FNB to only CFNC, we found no significant differences in either preoperative opioid use (*p* = 0.146), intraoperative opioid use (*p* = 0.119), postoperative use at the recovery room (*p* = 0.225) and postoperative use after 48 h (*p* = 0.422).

**Table 4:** Postoperative outcomes

	FNB (*N* = 118)	CFNC (*N* = 178)	No loco-regional anaesthesia (*N* = 112)	*p* value
Per hour opioid use				
Preoperatively in ml *M (SD)*	0.49 (2.2)	0.23 (0.38)	0.18 (0.30)	0.106
Intraoperatively in ml *M (SD)*	17.5 (9.6)	15.5(10.7)	19.7 (12.5)	**0.007**
Postoperatively at the recovery room *M (SD)*	0.36 (1.1)	0.37 (0.74)	0.93 (1.5)	** < 0.001**
Postoperatively 2 days after surgery *M (SD)*	0.05 (0.11)	0.06 (0.15)	0.1 (0.17)	**0.015**
Total opioid use				
Preoperatively in ml *M (SD)*	7.7 (45.9)	6.4 (10.5)	2.8 (4.0)	0.328
Intraoperatively in ml *M (SD)*	31.1 (13.6)	28.6 (15.9)	34.5 (16.2)	**0.007**
Postoperatively at the recovery room *M (SD)*	1.2 (3.4)	1.9 (3.4)	3.3 (4.5)	** < 0.001**
Postoperatively 2 days after surgery *M (SD)*	2.1 (5.1)	3.1 (7.1)	3.3 (6.9)	**0.015**
Complications				
Falls *n* (%)	2 (1.7)	4 (2.3)	0 (0)	0.675
Delirium *n* (%)	15 (12.7)	20 (11.4)	8 (7.1)	0.354
Anaemia *n* (%)	30 (25.4)	64(36.4)	39(34.8)	0.126
Urinary tract infection *n* (%)	9 (7.6)	9(5.1)	2(1.8)	0.122
CVA *n* (%)	0(0.0)	0(0.0)	1(0.9)	0.268
Myocardial Infarction *n* (%)	0(0.0)	1(0.6)	2(1.8)	0.270
Decubital ulcer *n* (%)	0(0.0)	0(0.0)	1(0.9)	0.268
Wound infection *n* (%)	2(1.1)	1(0.8)	0(0.0)	0.540
Patients with a complicated course *n* (%)	50(42.2)	88(50)	49(43.8)	0.430
Mortality				
30-day mortality	10(8.5)	14(8.0)	14(12.5)	0.409
90-day mortality	14(11.9)	32(18.4)	17(15.2)	0.317
1-year mortality	21(17.8)	58(33.3)	32(28.8)	**0.013**
Total HLOS in days *M (SD)*	9.4 (5.0)	9.3 (4.7)	8.8 (3.9)	0.590

*n* number of patients, *M* Mean, *SD* Standard Deviation, *HLOS* hospital length of stay, *FNB* femoral nerve block, *CFNC* continuous femoral nerve catheter, *CVA* cerebral vascular accident

#### CFNC group

In total, mean preoperative opioid use was 6.4 mg (SD 10.5; 95% CI 4.9–8.0). Intraoperative opioid use was 28.8 mg (SD 15.9; 95% CI 26.3–31.0) and opioid use at the recovery room and after 48 h was 1.9 mg (SD 3.4; 95% CI 1.4–2.4) and 3.1 mg (SD 7.1; 95% CI 2.0–4.2). Postoperative opioid use at the recovery room and intraoperative opioid use were significantly lower than the group that did not receive LRA (recovery room: *p* < 0.001; intraoperative: *p* = 0.007).

#### No intervention group

Preoperative opioid use was 2.8 mg (SD 4.0; 95% CI 2.1–3.6), intraoperative opioid use was 34.5 mg (SD 16.2; 95% CI 31.5–37.5). The intraoperative opioid use was significantly higher than the intervention groups (*p* = 0.007). Postoperatively, opioid use at the recovery room was 3.3 mg (SD 4.5; 95% CI 2.4–4.1) and opioid use 48 h after surgery was 3.3 mg (SD 6.9; 95% CI 3.2–6.3). Postoperatively, patients used significantly more opioids at the recovery room and 48 h after surgery than the intervention groups (recovery room: *p* < 0.001; after 48 h: *p* = 0.015). Median outcomes on opioid use are shown in supplement 1.

### Clinical outcomes

#### Complications

*Falls:* We compared the number of patients that experienced a postoperative fall 48 h after admission to intervention and found no significant difference between the groups (*p* = 0.675).

*Delirium:* Observing the total of patients who experienced delirium, we found a non-significant difference between the groups (*p* = 0.354).

*Anaemia*: In the CFNC group, 64 patients had anaemia (36.4%). No significant difference was found between the intervention groups (*p* = 0.126).

*Urinary tract infections:* FNB patients had the highest percentage of urinary tract infections (7.6%), no significant differences were found between the intervention groups (*p* = 0.122).

*CVA:* 1 patient from the no-LRA group had a CVA (0.9%). No significant difference between groups was found (*p* = 0.268).

*Myocardial infarction:* MI was observed in three patients, two of which were in the no-LRA group. (*p* = 0.270).

*Decubital ulcer*: In total, one patient out of the no-LRA group had a decubital ulcer. (0.9%; *p* = 0.268).

*Wound infection*: 3 patients suffered from a wound infection, 2 of which were in the FNB group (1.1%, *p* = 0.540).

Total number of patients with a complicated course:

In the FNB group, 50 patients (42.2%) had a complicated course, in the CFNC group 88 patients (50%) and in the no-LRA group 49 patients (43.8%). No significant differences were found when comparing the intervention groups (*p* = 0.430).

#### HLOS

When comparing HLOS between patients that received LRA and patients that did not receive any LRA, no significant difference was found. (*p* = 0.590).

Time from admission of LRA to surgery:

Regarding time from admission to surgery, we found that patients in the CFNC group had a significantly longer time than patients in the FNB group (10:50; SD 08:11) vs (1:52; SD 4:39; *p* < 0.001).

Time spent at the recovery room:

A difference was found concerning time spent at the recovery room. Patients who received CFNC spent most time at the recovery room, namely 5 h and 36 min (SD 4:33) and patients who received FNB spent the least amount of time at the recovery room (4:02 (SD 2:44)). Patient who received conventional therapy (no LRA) spent an average of 4:43 h at the recovery room (SD 4:11). Patients with CFNC spent significantly more time at the recovery room (*p* = 0.006).

Mortality:

In the no-LRA group, 30-day mortality was highest (*n* = 14, 12.5%). In the CFNC group, 90-day mortality was highest (*n* = 32, 18.4%). No significant differences were found in 30-day mortality (*p* = 0.409) and 90-day mortality (0.317). In the CFNC group, 1-year mortality was highest (*n* = 58, 33.3%). There was a significant difference in 1-year mortality between the intervention groups (*p* = 0.013). When comparing patients who received any form of LRA (FNB or CFNC) to patients who did not receive LRA, no significant difference in 1-year mortality was found.

### Multivariate analysis

Multivariate tests were used to predict pre-, intra- and postoperative opioid use from intervention, ASA, gender, operation duration (if applicable) and age.

#### Preoperative

A significant regression equation was found (F (4, 398) = 2.800, *p* = 0.026), with an R^2^ of 0.018. Patients’ predicted preoperative morphine use is equal to 0.842 + 2.472 (intervention) + 1.163 (ASA) -0.050 (age) + 0.506 (gender), where intervention is coded as 0 = no LRA 1 = FNB or CFNC, ASA is coded as 1 = ASA 1, 2 = ASA 2, 3 = ASA 3, 4 = ASA 4, gender is coded as 1 = female, 2 = male and age is coded in years. Patients’ preoperative morphine use increased with 1.163 per ASA classification and it decreased with 0.050 per year of age. Females used 0.506 times more morphine than man. *Patients without LRA used 2.472 times less morphine than patients with LRA.*

#### Intraoperative

At the recovery room: A significant regression equation was found (F (5, 395) = 15.505, *p* < 0.001), with an R^2^ of 0.164. Patients’ predicted intraoperative morphine use is equal to 51.328 − 4.919 (intervention) + 0.257 (ASA) + 0.106 (operation duration) -0.277 (age) -2.124 (gender), where intervention is coded as 0 = no LRA 1 = FNB or CFNC, ASA is coded as 1 = ASA 1, 2 = ASA 2, 3 = ASA 3, 4 = ASA 4, operation duration is coded in minutes, gender is coded as 1 = female, 2 = male and age is coded in years. Patients’ postoperative morphine use at the recovery room increased with 0.257 per ASA classification and increased with 0.106 per minute of surgery. It decreased with 0.277 per year of age and males used 2.124 times more morphine than man. *Patients without LRA used 4.919 times more morphine than patients with LRA.*

#### Postoperative

- At the recovery room: A significant regression equation was found (F (5, 395) = 5.080, *p* < 0.001), with an R^2^ of 0.060. Patients’ predicted postoperative morphine use at the recovery room is equal to 8.139 -1.689 (intervention) + 0.489 (ASA) + 0.006 (operation duration) -0.063 (age) + 0.084 (gender), where intervention is coded as 0 = no LRA 1 = FNB or CFNC, ASA is coded as 1 = ASA 1, 2 = ASA 2, 3 = ASA 3, 4 = ASA 4, operation duration is coded in minutes, gender is coded as 1 = female, 2 = male and age is coded in years. Patients’ postoperative morphine use at the recovery room increased with 0.489 per ASA classification and increased with 0.006 per minute of surgery. It decreased with 0.063 per year of age and females used 0.084 times more morphine than man. *Patients without LRA used 1.689 times more morphine than patients with LRA.*
- After 48 h: A significant regression equation was found (F (5, 395) = 5.615, *p* < 0.001), with an R^2^ of 0.055. Patients’ predicted postoperative morphine use after 48 h is equal to 12.236 -2.076 (intervention) + 1.950 (ASA) + 0.010 (operation duration) − 0.132 (age), − 1.195 (gender) where intervention is coded as 0 = no LRA 1 = FNB or CFNC, ASA is coded as 1 = ASA 1, 2 = ASA 2, 3 = ASA 3, 4 = ASA 4, operation duration is coded in minutes, gender is coded as 1 = female, 2 = male and age is coded in years. Patients’ postoperative morphine use after 48 h increased with 1.950 per ASA classification and increased with 0.010 per minute of surgery. It decreased with 0.132 per year of age and males used 1.195 times more morphine. *Patients without LRA used 2.076 times more morphine than patients with LRA.*

## Discussion

Hip fractures in elderly patients are a common health problem and optimal pain management is needed to prevent complications and opioid-related side effects and to optimize perioperative care. This study compared FNB and CFNC to no use of LRA. We found significantly lower opioid use intraoperatively, postoperatively at the recovery room and postoperatively after 48 h in patients who had received LRA. Moreover, the use of LRA was a significant predicter for postoperative opioid use, both at the recovery room and after 48 h.

To our knowledge, this is the first and only study that compares both FNB and CFNC to no LRA and that also investigates (long-term) mortality. We found no significant differences between the two different types of LRA. Previous literature focused mostly on either CFNC or FNB. In earlier randomized controlled trials, a decrease in opioid use in patients with LRA has been reported. [11, 15, 16]. However, these studies had a follow-up of only 24 h, they did not measure pre-, intra-, and operative opioid use and they included a relatively small number of patients. In a retrospective study from 2017, which was conducted in Stanford, opioid use and opioid-related side effects in geriatric hip fracture patients who received a CFNC was evaluated and compared to patients who did not receive LRA [7]. A significant decrease of opioid-related side effects in patients who had received CFNC was found, which is consistent with our findings. The study from Helsø et al. compared patients with continuous femoral block with patients without continuous femoral nerve block [23]. 456 patients were included, and they found a decrease in total opioid use as well. Nonetheless, this difference was insignificant, which differs from our results. This may be due to the unevenly distributed number of patients in the study groups (366 patients with continuous femoral block vs. 90 patients without continuous femoral block). Moreover, they had to exclude a large group of patients due to incomplete data, which may have caused a type II error. Another study from the Netherlands, from 2019, compared elderly hip fracture patients with a single-shot FNB to those without LRA. They also found a significant decrease in opioid use and pain (as measured by VAS-score). [24] This is similar to our results.

Previous literature reports as a possible side-effect, FNB may cause a motor block, which leads to a higher risk of in-hospital falls [25]. Hence, we collected data on postoperative falls. Nonetheless, we found no significant difference between the groups. The aforementioned study from Helsø evaluated falls and found no significant differences in falls between the two intervention groups as well, which is conform our findings [23]. In both studies, these findings may be due to a lack of reporting, which may have caused an underestimation of the total number of falls. In addition, they also observed HLOS, which did not differ between the two groups either. HLOS was reported in other previous literature as well, none of which found a significant difference. A recent Cochrane review from November 2020, reviewed peripheral nerve blocks in the treatment of hip fractures. [26] They found no significant differences in short-term mortality, which is comparable to our results. However, they did not compare FNB to CFNC, only LRA to no LRA. Similar to our results, the study from Guay also found no significant differences in MI in hip fracture patients with- or without peripheral nerve blocks [26]. Regarding 1-year mortality, we found that patients who received CFNC had significantly higher mortality rates. This could have been caused by the higher proportion of ASA 3 and ASA 4 patients in that group. No previous literature was found on 1-year mortality between FNB and CFNC. Literature comparing mortality between CFNC and FNB together with no LRA was found and did not show a significant difference, which is similar to our results [26–28]. A recent RCT from Sweden researched complications in patients with FNB compared to those with conventional pain management, they found no significant differences in urinary tract infections and anaemia, which is the same as our results [29]. Even though we would expect a higher rate of delirium in patients without LRA, the number of patients with delirium did not differ significantly between the two groups. Likewise, a previous study from 2015 and the previously named study from Sweden reported no differences in delirium [29, 30]. This raises the question if LRA protects patients from developing delirium. In previous studies, though, they did show a trend that suggests LRA prevents patients from having a delirium but we did not find such a trend [31]. Most likely, this is due to the abovementioned lack of adequacy in reporting of data. Conform previous literature, this study also observed no significant difference in the rate of patients with a complicated course.

Remarkably, there was a high percentage of patients who received general anaesthesia, though usually spinal anaesthesia is preferred in hip fracture surgery. This was due to hospital guidelines and surgeon preferences. A Cochrane review found no significant differences between both techniques aside from a lower risk of deep venous thrombosis in patients without prophylactic anticoagulation therapy who received spinal anaesthesia [32]. We also found a significantly longer time between admission of the intervention to surgery in CFNC patients, even though elongated time to surgery has been proven to increase mortality and other complications [33–35].

Strengths of this study include the fact that it includes both CFNC and a single-shot FNB concurrently. Furthermore, the observation period is relatively long, and we obtained data from pre-, intra-, and postoperative periods. In addition, this is one of the few studies that contained data on (long-term) postoperative mortality. Data collection was performed by three independent researchers, which makes it reliable. However, little data in the Electronical Medical Record (EMR) were available for outcomes such as falls, delirium and other complications which suggests data may have been incomplete. There are several other limitations to this study. First, it has a retrospective design which causes known and unknown sorts of bias. Second, the decision on type and use of LRA was not protocolized. Instead, it was mostly operator- and patient dependent. Patients with a local infection or with a severe coagulopathy were not deemed suitable to receive LRA. This may have posed a selection bias. Third, there was no uniformity in the dose of anaesthetic that was administered to the catheter or the block. Therefore, an omitted variable bias may have occurred. However, this is explained by the fact that the design of this study was a quality assessment of everyday clinical practice. Last, incorrect placement may have caused an overestimation in the reporting of pain.

## Conclusion

This retrospective cohort study shows that LRA in the form of FNB and CFNC causes a significant decrease in postoperative opioid consumption and that LRA is a significant predictor for postoperative opioid use. It also showed that differences in single-shot FNB or CFNC were minimal and non-significant. Nonetheless, there were no significant differences in clinical outcomes such as HLOS, complications, short-term mortality and postoperative falls. Therefore, we suggest that use of LRA should be incorporated in the perioperative treatment of elderly patients with a hip fracture: we recommend FNB for patients with minor coagulation disorders and CFNC for patients without any coagulation disorders and without a local infection. We do not recommend use of LRA in patients with severe coagulopathy or in patients who have a history of a femoral-popliteal bypass. For future research, we recommend to further investigate the 1-year mortality rates between LRA and no LRA and the difference between CFNC and FNB.

## Supplementary Information

Below is the link to the electronic supplementary material.

Supplementary file1 (DOCX 7117 kb)
