# Supplementary material for: The impact of loco-regional anaesthesia on postoperative opioid use in elderly hip fracture patients: an observational study
Source: Eur J Trauma Emerg Surg. 2021 May 7;48(4):2943–52. doi: 10.1007/s00068-021-01674-4 (PMC9360082; doi:10.1007/s00068-021-01674-4)
Supplement: Supplementary file 1 — Supplementary file1 (DOCX 7117 kb) [file 68_2021_1674_MOESM1_ESM.docx]

**Appendix**

**Supplement 1:** Medians

|  | FNB (N=118) | CFNC (N=178) | No locoregional anaesthesia (N=112) | P-value |
| --- | --- | --- | --- | --- |
| **Per hour opioid use** |  |  |  |  |
| Preoperatively in ml *Md (IQR)* | 0.05 (0.0;0.37) | 0.10 (0.0;0.30) | 0.07 (0.0;0.29) | 0.602 |
| Intraoperatively in ml *Md (IQR)* | 16.12 (12.6;22) | 14.8(8.5;20.5) | 18.4 (12.5;24.4) | 0.125 |
| Postoperatively at the recovery room *Md (IQR)* | 0.0 (0.0;0.21) | 0.0 (0.0;0.45) | 0.33 (0.0;1.08) | **<0.001** |
| Postoperatively 2 days after surgery *Md (IQR)* | 0.0(0.0;0.04) | 0.0(0.0;0.08) | 0.04 (0.0;0.13) | **<0.001** |
|  |  |  |  |  |
| **Total opioid use** |  |  |  |  |
| Preoperatively in ml *Md (IQR)* | 1 (0.0;5.25) | 2.5(0.0;7.5) | 2.0(0.0;4.9) | 0.89 |
| Intraoperatively in ml *Md (IQR)* | 30 (22.5;40) | 30(20;40) | 35 (25;45) | 0.148 |
| Postoperatively at the recovery room *Md (IQR)* | 0.0(0.0;0.0) | 0.0(0.0;2.5) | 2.0(0.0;5.0) | **<0.001** |
| Postoperatively 2 days after surgery *Md (IQR)* | 0.0(0.0;2.0) | 0.0(0.0;4.0) | 2.0(0.0;6.0) | **<0.001** |
| n: number of patients. Numbers are noted in percentages of the total number of study patients (missing values were excluded from analysis). M: Mean SD: Standard Deviation. HLOS: hospital length of stay. FNB: femoral nerve block. CFNC: continuous femoral nerve catheter | | | | |

**Supplement 2: Figure A1:** Boxplot of preoperative morphine use


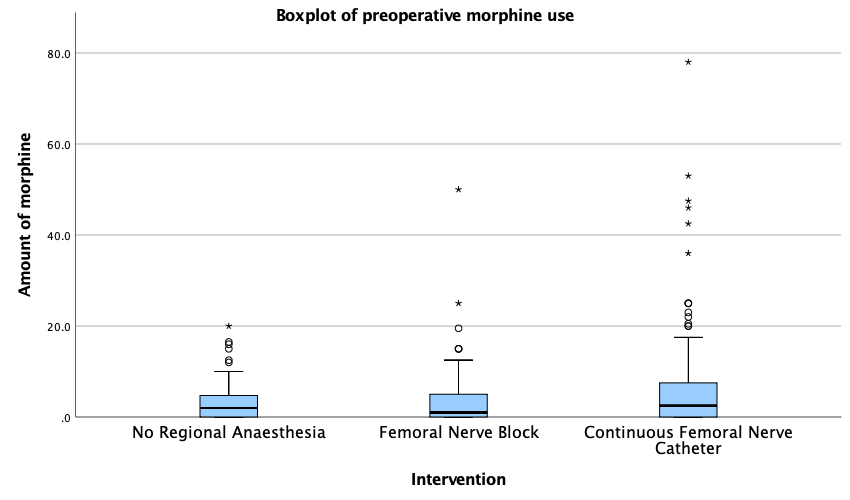


**Supplement 3:** Boxplot of intraoperative morphine use


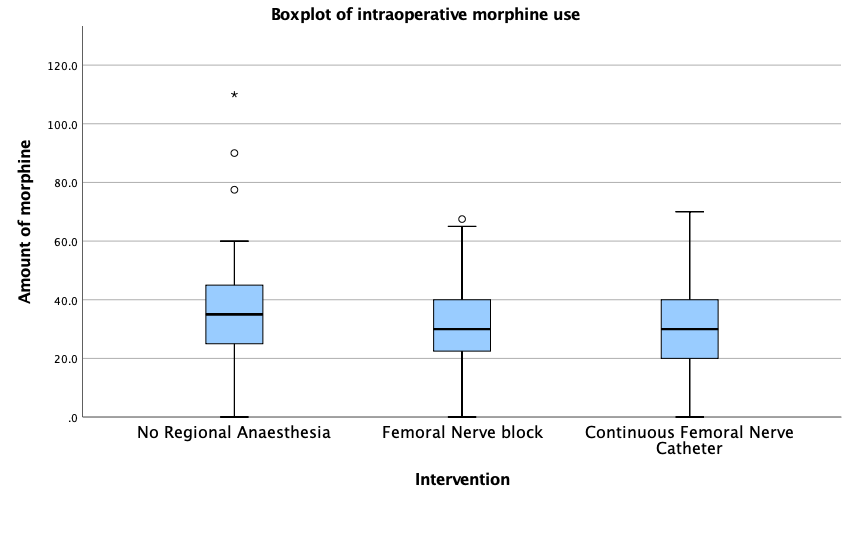


**Supplement 4: Boxplot of postoperative morphine use at the recovery room**

**
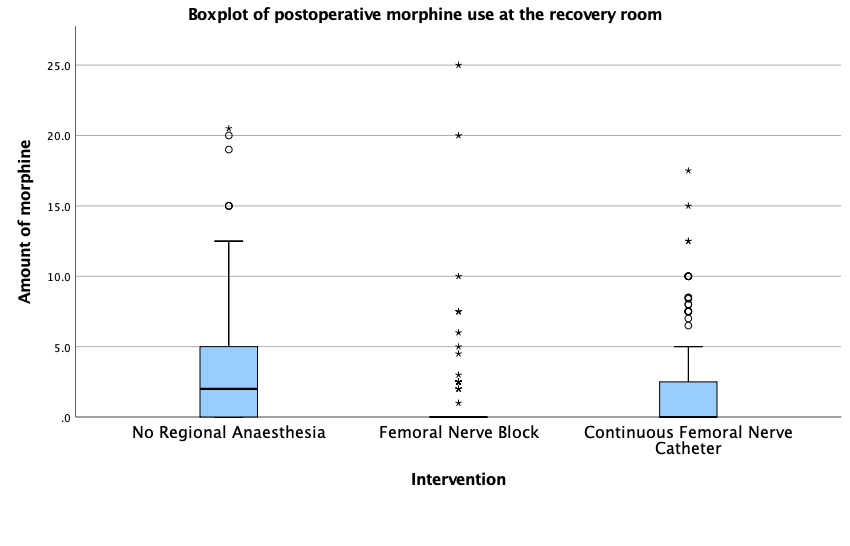
**

**Supplement 5: Boxplot of postoperative morphine use after 48 hours**

**
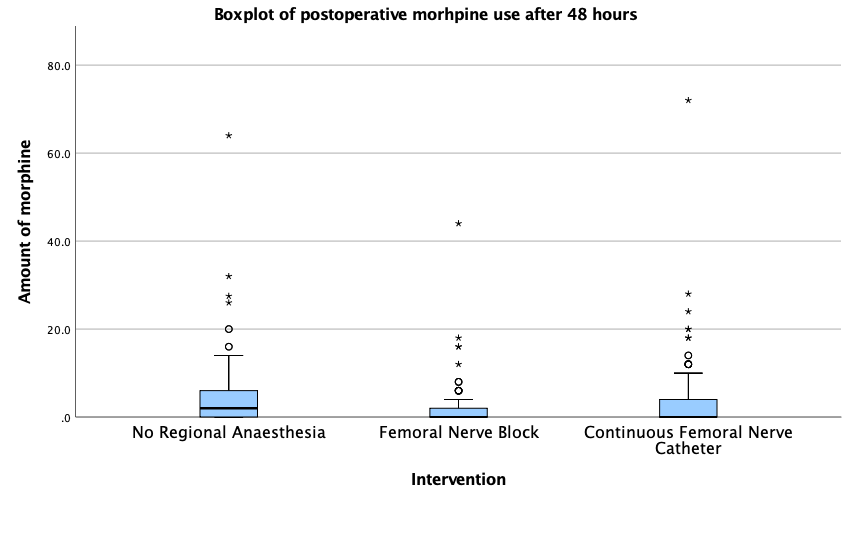
**
